# Long Noncoding RNAs in Pathological Cardiac Remodeling: A Review of the Update Literature

**DOI:** 10.1155/2019/7159592

**Published:** 2019-07-01

**Authors:** Huan Zhou, Bin Wang, Ying-xi Yang, Qiu-jin Jia, Ao Zhang, Zhong-wen Qi, Jun-ping Zhang

**Affiliations:** ^1^Traditional Chinese Medicine Department, Affiliated Hospital of Nankai University, Tianjin, China; ^2^Cardiology Department, First Teaching Hospital of Tianjin University of Traditional Chinese Medicine, Tianjin, China; ^3^Endocrinology Department, First Teaching Hospital of Tianjin University of Traditional Chinese Medicine, Tianjin, China; ^4^Epidemiology, College of Global Public Health, New York University, New York, USA

## Abstract

Cardiac remodeling is a self-regulatory response of the myocardium and vasculature under the stressful condition. Cardiomyocytes (CMs), vascular smooth muscle cells (VSMCs), endothelial cells (ECs), and cardiac fibroblasts (CFs) are all involved in this process, characterized by change of morphological structures and mechanical/chemical activities as well as metabolic patterns. Despite current development of consciousness, the control of cardiac remodeling remains unsatisfactory, and to further explore the underlying mechanism and seek the optimal therapeutic targets is still the urgent need in clinical practice. It is now emerging that long noncoding RNAs (lncRNAs) play key regulatory roles in these adverse responses: lncRNA TUG1, AK098656, TRPV1, GAS5, Giver, and Lnc-Ang362 have been indicated in hypertension-related vascular remodeling, H19, TUG1, UCA1, MEG3, APPAT, and lincRNA-p21 in atherosclerosis (AS), and HIF1A-AS1 and Lnc-HLTF-5 in aortic aneurysm (AA). In addition, Neat1, AK139328, APF, CAIF, AK088388, CARL, MALAT1, HOTAIR, XIST, and NRF are involved in postischemia myocardial remodeling, while Mhrt, Chast, CHRF, ROR, H19, Plscr4, and MIAT are involved in myocardial hypertrophy, and MALAT1, wisper, MEG3, and H19 are involved in extracellular matrix (ECM) reconstitution. Signaling to specific miRNAs by acting as endogenous sponge (ceRNA) was the main form that regulates the target gene expression during cardiac remodeling. This review will underline the updates of lncRNAs and lncRNA-miRNA interactions in maladaptive remodeling and also cast light on their potential roles as therapeutic targets, hoping to provide supportive background for following research.

## 1. Introduction

Cardiovascular diseases, especially coronary heart disease (CHD) and heart failure (HF), remain the leading cause of mortality worldwide, despite a dramatic reduction due to current therapeutic advances [1, 2]. Generally, acute ischemic events can be rapidly improved by timely revascularization, while progressive cardiac remodeling is becoming the new clinical puzzle. Cardiac remodeling, which mainly refers to rearrangement of normal structures, is a chronic maladaptive process characterized by vascular dysfunction, myocardial hypertrophy, apoptosis, necrosis, ventricular dilatation, and fibrosis [3]. Up to now, the underlying mechanism of this process has not been completely elucidated; several pathogeneses are involved: dysregulated neurohumoral stimulation, ischemia-related damage, increased hemodynamic overload, extracellular matrix (ECM) anomalies, immunological activation, accelerated cell apoptosis, and genetic mutations [4]. Agents targeting mitochondrial function/nerve-endocrine-immunity (NEI) network or utilization for ischemic conditioning/stem cell transplantation has been proved to partly alleviate adverse remodeling [5–7], but the role is still limited, thus exploring new biomarkers for diagnosis or as novel therapeutic targets for pathological remodeling needs further efforts. At this point, though, emerging data have suggested a fundamental role for noncoding RNAs (ncRNAs) in remodeling-related cardiovascular diseases including atherosclerosis (AS), hypertension, aneurysm, postinfarct myocardial remodeling, and hypertrophic cardiomyopathy.

Long noncoding RNAs (LncRNAs), a kind of functional RNA molecules with the length of over 200 nucleotides, have once been regarded as the “noise” of genome transcription because of their deficiency in protein-coding process [8], but they now gained much attention in various fields like cancer and cardiocerebrovascular disease with the advancement of transcriptome program and chip technology. Plenty of evidence showed that lncRNAs are capable of regulating the occurrence and development of certain disorders. For instance, lncRNA H19 has been reported to be upregulated in atherosclerotic patients; by signaling to unique miRNAs or proteins, it may participate in multiple processes like vascular smooth muscle cell (VSMC) apoptosis, inflammation activation, and myocardial cell necrosis, thus deteriorating AS progression and ischemia-reperfusion (I/R) injury [9–13]. According to the update studies, lncRNAs not only served as diagnostic markers in cardiovascular disease, but also exhibited potentials for therapeutic applications [14]. Therefore, we provide a systematic perspective based on the regulatory roles of lncRNAs and discuss the challenges and possible applications of lncRNAs in cardiac remodeling.

## 2. LncRNAs: What Are They?

### 2.1. An Overview on Molecular and Biological Roles of LncRNAs

It is known that only 1.5% of the human genome is of protein-coding potential, and the remaining majority is illustrated to have no or very little protein-coding function [15]. LncRNAs, belonging to a class of ncRNAs with the length of more than 200 nucleotides, are now reported to have strong epigenetic regulation potentials. With the progress of high-throughput sequencing technology, thousands of eukaryotic lncRNAs are continually being found [16], and their expression profile seems to be cell-type specific and their subcellular localization in nucleus or cytoplasm is well-arranged [17, 18].

To understand the diverse species of lncRNAs more accurately, there are several convenient classification criteria. For example, according to genomic distribution, lncRNAs can be classified into 5 categories including sense lncRNAs, antisense lncRNAs, bidirectional lncRNAs, intronic lncRNAs, and intergenic lncRNAs, which were closely related to their stabilities [19]. In regard to biological functions, nuclear compartmentalization, genomic imprinting, X chromosome inactivation, cell fate specification, and RNA splicing were mainly involved. Here, we will summarize the current findings as follows.

#### 2.1.1. Molecular Functions

**Figure 1:**
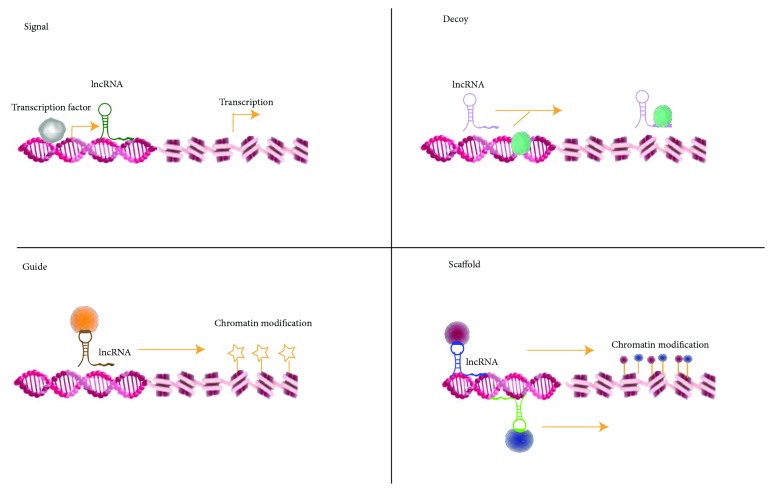
Schematic Diagram of Basic LncRNA Mechanism.

Similar to the protein-coding RNAs, lncRNAs have unique subcellular distribution that is crucial for functions, predominantly in nucleus and partially in the cytoplasm. The nuclear lncRNAs mainly perform transcriptional regulatory effects via guiding chromatin modifiers [20]. Others in cytoplasm were discovered to modulate mRNA translation or impact protein trafficking. According to the archetypes of molecular functions, lncRNAs were classified into 4 main categories: signals, decoys, guides, and scaffolds. As shown in Figure 1, briefly, among them are (1) signal lncRNAs, which could modulate gene expression in a time and space manner, (2) decoy lncRNAs, which could titrate transcription factors away from chromatin or titrate miRNAs away from their targets (acting as miRNA sponge), (3) guide lncRNAs, which serve as molecular chaperons, which can recruit chromatin-modifying enzymes to target genes, either in neighboring (cis) or distant location (trans), and (4) scaffold lncRNAs, which act as molecular scaffold, accumulating proteins to form ribonucleoprotein complexes and then promoting histone modification [21–23].

Schematic diagram of basic LncRNA mechanism: nuclear-localized lncRNAs regulate gene expression basically in 4 modes including (1) signal, where lncRNAs expression can reflect actions of transcription factors (gray ovals), (2) decoy, where lncRNAs can sequester transcription factors/protein complex, and (3) guide, where lncRNAs can guide transcription factors/protein complex to specific target site and bring together multiprotein complexes as (4) scaffold.

#### 2.1.2. Biological Functions

For biological functions, lncRNAs have been demonstrated to play multilayer roles such as the following: (1) nuclear compartmentalization: lncRNAs may contribute to regulating subnuclear structure such as paraspeckle formation; (2) chromatin modification: lncRNAs could participate in chromatin modification by interacting with epigenetic regulators and directing them into specific chromatin regions; lncRNA Kcnq1ot1, Air, and HOTAIR were all available examples [24, 25]; (3) RNA splicing: RNA splicing is a process that transforms the precursor messenger RNA (pre-mRNA) transcript into a mature messenger RNA (mRNA); (4) X chromosome inactivation: lncRNAs are also able to regulate X chromosome inactivation. For instance, X-inactive specific transcript (XIST) is known to silence hundreds of genes on the X chromosome in female somatic cells [26]. The other detailed functions will not be clarified here, but they also merit concern.

## 3. The Regulatory Role of LncRNA in Cardiac Remodeling

### 3.1. LncRNAs in Vascular Remodeling

The vessel wall is an integrated organ, containing endothelial cells (ECs), VSMCs, and also matrix components. Its function or structure is sensitive to variety of stimuli, many of which might drive physiopathologic changes. The dynamic adaptive process is defined as ‘vascular remodeling.' Vascular remodeling is accepted as the main pathological basis of hypertension and atherosclerosis as well as arterial aneurysm (AA), etc., which is usually characterized by increased vascular resistance, thickened intima, segmental stenosis, or aneurysmal dilatation. VSMC dysfunction serves as the foremost cellular basis for these adverse processes. Furthermore, the regulation of VSMCs is demonstrated to be quite involved with lncRNAs [27] (Table 1).

#### 3.1.1. Hypertension-Related Vascular Remodeling

LncRNAs such as taurine upregulated 1 (TUG1) [28], AK098656 [29], transient receptor potential vanilloid type 1 (TRPV1) [30], growth block specificity 5 (GAS5) [31], growth factor-and proinflammatory cytokine-induced vascular cell-expressed lncRNA (Giver) [32], and Lnc-Ang362 [33] are newly demonstrated participators in hypertension-related remodeling through regulating VSMC behaviors. Study shows that lncRNA TUG1 was highly concentrated in aorta of spontaneously hypertensive rats (SHR). The overexpressed TUG1 is not just a biomaker, but an essential actor via sponging miR-145-5p to promote the migration and proliferation of SHR-VSMCs, subsequently causing Wnt/*β*-catenin pathway activation [28]. Similarly, Jin et al. revealed that lncRNA-AK098656 mainly of VSMC origin was markedly upregulated in the plasma of hypertension patients compared to healthy ones. AK098656-overexpressing transgenic rats would spontaneously progress to hypertension, presenting increased media thickness and narrowed arterial lumen. The promotion of VSMC phenotypic switch by directly binding to the SM-specific contractile proteins and inducing their degradation undertook the underlying mechanism [29]. LncRNA GAS5, mainly expressed in ECs/VSMCs, was also suggested to participate in hypertension via modulating multiple links such as EC proliferation, VSMC phenotype conversion, and EC-VSMC communication by *β*-catenin signaling; knockdown of GAS5 could aggravate microvascular dysfunction [31]. Giver, the nuclear enriched transcript, was found to dramatically be overexpressed in arteries from untreated hypertensive patients and identified as a novel regulator of AngII-induced VSMC dysfunction by microarray analysis. Mechanically, it might promote oxidative stress, VSMC proliferation, and vascular inflammation via altering the state of chromatin—negatively regulating its neighboring gene Nr4a3 expression [32].

#### 3.1.2. Atherosclerotic Vascular Remodeling

Plenty of studies have been published on lncRNA-mediated atheromatous plaque development via regulating responses of vessel cells [34]. Through database retrieval, NONCODE, H19, ANRIL, and CDKN2B-AS1 were identified as AS-related lncRNAs [9]. Among them, LncRNA H19 is the hotspot in recent decades, which not only acts as the potential serum marker of CHD [35, 36], but also largely takes part in the progression of AS. Huang et al. demonstrated that lentivirus-mediated H19-forced expression promotes VSMC proliferation and inhibits its apoptosis in vitro, and if in the ischemic stroke mouse model, an increased plaque size was detected with H19 overexpression but dramatically diminished while silencing. Upregulating the expression of acid phosphatase 5(ACP5) at posttranscriptional level is the key mechanism by which H19 contributes to the harmful performance [9]. Zhang et al. have also reported the roles of H19 in AS previously. The interpretation still attributed to regulation of the imbalance of VSMC proliferation and apoptosis. Knockdown of H19 efficiently suppressed proliferation and facilitated apoptosis in ox-LDL-treated human aorta VSMCs by blocking Wnt/*β*-catenin pathway, thus alleviating intimal thickening [11]. Besides, elevated expression of H19 was as well detected in ox-LDL-stimulated Raw264.7 cells, which played roles in promoting lipid accumulation and proinflammatory factors release [12]. Thereby, the approach to silence H19 expression might turn into a promising application in preventing AS, although the deep mechanism is still imperative in vivo.

LncRNA TUG1, as mentioned above, is a vital regulator in modulation of VSMC behaviors. Recent studies have likewise explored its impacts on the advancement of AS. Results showed TUG1 was highly expressed in the serum of patients suffering from AS as well as in the plaque tissue of ApoE-/-mice. Knockdown of TUG1 led to suppressed inflammatory response and attenuated atherosclerotic lesion. The abnormal proliferation of VSMCs [37], together with excessive apoptosis of ECs [38] and macrophages [39] induced by TUG1-miRNA (miR-21/133a/26a) interactions, took the main responsibility.

Urothelial carcinoma-associated (UCA1) [40] and maternally expressed gene 3 (MEG3) [41] are regarded as two types of protective lncRNAs under the atherogenic conditions, both of which could perform as endogenous sponge of miR-26a, and induce a modified expression of the target proteins (e.g., PTEN and Smad1).

Atherosclerotic plaque pathogenesis associated transcript (APPAT), just as its name, is an important predictor of disease progression. Significant decrease of APPAT was detected in coronary artery samples with severe stenosis and high-risk individuals. The potential mechanism is partly attributed to the influence of VSMC phenotype shift via signaling to special miRNAs (e.g., miR-647/135b) [14].

Besides, long intergenic noncoding RNA-p21 (lincRNA-p21) had been identified as another beneficial regulator against AS. Levels of lincRNA-p21 were markedly downregulated in plaque lesions of ApoE-/-mice model as well as in coronary artery tissue of CHD patients, and lentivirus-si-lincRNA-P21 injected into the injury site induced dramatical neointimal hyperplasia [42].

Nuclear paraspeckle assembly transcript 1 (Neat1) was reported to promote intima thickening or even vascular occlusion by modulating the phenotype conversion of VSMCs. Neat1 knockout mice showed suppressed neointima formation after vascular injury, and the upregulation of SM-contractile gene was the main mechanism [43].

#### 3.1.3. Aortic Aneurysm

Aortic aneurysm is a life-threatening pathological condition with the possibility to rupture when silently progressing to the advanced state, generally characterized by VSMC loss, medial degeneration, and bulging of the vessel wall. Currently, no specific pharmacological approaches exist that could slow down the progression and risk of aneurysm rupture, except for surgical intervention. With advances of molecular research, the participation of lncRNAs has been gradually concerned. LncRNA-related VSMC dysfunction and ECM degradation were suggested as potential mechanisms according to recent data. Microarray profile analysis revealed that hundreds and thousands of lncRNAs are involved in human thoracic aortic aneurysm (TAA) development, such as Lnc-HLTF-5 [44], HIF1A-AS1, RP11-465L10.10, and CTD-2184D3.5 [45], which might play roles in regulating the expression of MMP-9 in aortic tissue and then facilitate the expansionary remodeling. HIF1alpha-antisense RNA 1 (HIF1A-AS1) was the first reported lncRNA participating in TAA pathogenesis [46]. Increased expression of HIF1A-AS1 was usually found in plasma of TAA patients, knockdown of which led to attenuated apoptosis of VSMCs by suppressing caspase-3/8 expression [47]. LincRNA-p21 was another reported lncRNA associated with TAA. Upregulated expression of lincRNA-p21 is found both in aortic media and in blood samples in established TAA patients. LincRNA-p21 forced expression might hinder proliferation and promote apoptosis of VSMCs, thus thinning the media [48]. The discovery of the association between lncRNAs and TAAs might provide potential diagnostic biomarkers or therapeutic targets for this occult but dangerous disease.

### 3.2. LncRNAs in Myocardial Remodeling

Myocardial remodeling, which consists of morphological, behavioral, metabolic, or electrical alteration, is a maladaptive response that is common in ventricular remodeling after MI or various types of cardiomyopathy. Myocardial remodeling is mainly manifested as loss of cardiomyocytes, cardiac hypertrophy, metabolic abnormalities, defective autophagy, electrical remodeling, and so on. Ischemia injury would promote the release of reactive oxygen species (ROS), resulting in dysfunction of the energy metabolism, or even death of cardiomyocytes. Persistent loss of cardiomyocytes may ultimately induce cardiac remodeling. With regard to cardiac hypertrophy, it is a compensative response to volume or stress overload stimuli, aiming at lowering the increased wall tension and maintaining the cardiac output, while persistent exposure of enhanced load would also accelerate cardiomyocytes loss, interstitial fibrosis, or even cardiac failure (Table 2).

#### 3.2.1. LncRNAs in Postischemia/Hypoxia Myocardial Remodeling

Up to now, by using high-throughput RNA sequencing, numerous lncRNAs have been found involved in the modulation of cardiac remodeling. In I/R condition, lncRNAs are reported to largely participate in myocardial autophagy (e.g., Neat1, AK139328, APF, CAIF, and AK088388), apoptosis (e.g., CARL, MALAT1, HOTAIR, UCA1, and XIST) and necrosis (e.g., NRF and H19), and then regulating the remodeling process. Neat1 [49] and AK139328 [50] were both demonstrated to be overexpressed in I/R-treated diabetic rat myocardial tissue; upregulated Neat1 and AK139328 might aggravate I/R injury via activating autophagy of cardiomyocytes, while inhibiting their expression significantly alleviates the damage. Autophagy promoting factor (APF), a novel regulator in autophagy, can induce autophagic cell death via signaling to miR-188-3p, thus augmenting myocardial infarction size [51]. Another characterized lncRNA termed cardiac autophagy inhibitory factor (CAIF) was nevertheless found to show opposite effects. Enforced expression of CAIF significantly attenuated autophagic death and infarction size induced by I/R injury, with the mechanism that p53/myocardin-mediated harmful autophagy was blocked [52]. Besides, in a hypoxia/reoxygenation (H/R) cardiomyocyte model, AK088388 was demonstrated to be highly expressed, which could competitively bind to miR-30a targeting beclin-1 and LC3-II expression, inducing autophagic injury; miR-30a mimics or siRNA-AK088388 however could maintain cardiomyocyte viability and reduce apoptosis [53].

Cardiac apoptosis-related lncRNA (CARL) was an early described apoptosis inhibitor of cardiomyocytes, which could suppress anoxia-induced mitochondrial fission and apoptosis by sponging miR-539 targeting prohibitin-2 (PHB2) [54]. Metastasis-associated lung adenocarcinoma transcript1 (MALAT1) is another highly expressed lncRNA in cardiac tissue during I/R injury, which may exacerbate cardiomyocyte inflammation and apoptosis by sponging miR-203 [55]. HOX antisense intergenic RNA (HOTAIR) was reported as a protective lncRNA, usually downregulated by hypoxia exposure or acute myocardial ischemia. HOTAIR overexpression markedly limited hypoxia/ischemia-induced myocardial apoptosis, whereas knockdown of HOTAIR severely accelerated apoptosis. Mechanically, the cardioprotective effect is partly based on HOTAIR-miR-125 or HOTAIR-miR-1 interactions [56, 57]. LncRNA UCA1 could be triggered reactively during I/R injury; overexpression of UCA1 by adenovirus transfection conferred significant myocardial protection by suppressing endoplasmic reticulum stress and ROS-induced cell apoptosis [58]. XIST was found to overexpress in cardiomyocytes after infarction, and the highly expressed XIST might facilitate apoptosis and inhibit proliferation by targeting miR-130a-3p [59].

In terms of myocardial necrosis, the lncRNA named necrosis-related factor (NRF) is worth mentioning. NRF facilitated the programmed necrosis of myocardial cells under I/R condition by the mechanism of sponging miR-873 expression and promoting RIPK1/RIPK3-induced necrotic cell death; knockdown of NRF could effectively antagonize necrosis and confer cardioprotection [60]. Except for NRF, H19 was also a participator. It regulated RIPK1/RIPK3-dependent myocardial cell necrosis by directly binding to miR-103/107 and further modulating the expression of FADD (fas-associated protein with death domain), the target gene of miR-103/107 [61].

#### 3.2.2. LncRNAs in Myocardial Hypertrophy

Myocardial hypertrophy is an adaptive morphological change to volume or stress overload stimuli, compensatory at the start but useless or harmful in the late stage. LncRNAs are emerging as new players in this pathophysiological process. Myosin heavy chain associated RNA transcript (Mhrt) is the first example of lncRNA which acts as chromatin remodelers and inhibits pathological cardiac hypertrophy. Mhrt has been confirmed to interact directly with Brg1 (the significant chromatin-remodeling factor in myocardial hypertrophy) and sequester Brg1 from its genomic DNA targets so as to maintain cardiac performance [62, 63]. According to cell fractionation experiment, cardiac hypertrophy-associated transcript (Chast) was indicated to be specifically upregulated in cardiomyocytes from transverse aortic constriction (TAC)-operated mice model and also in hypertrophic heart tissue from aortic stenosis patients. The mechanism by which it drives hypertrophy is involved in the negative regulation of pleckstrin homology domain-containing protein family M member 1(Plekhm1), the gene located on its opposite strand, thus hindering cardiomyocyte autophagy and facilitating hypertrophy [64].

Besides, unlike the chromatin- or gene-regulation effect of lncRNAs mentioned above, many other lncRNAs were reported to participate in cardiac hypertrophy by functioning as sponges of miRNAs, such as cardiac hypertrophy-related factor (CHRF) [65], ROR [66], H19 [67], Plscr4 [68], and myocardial infarction-associated transcript (MIAT) [69]. CHRF showed an antihypertrophic effect in vitro by suppressing miR-489/myeloid differentiation factor 88 (MyD88) signaling as an endogenous sponge. According to Jiang's study, lncRNA ROR was demonstrated as an impeller of cardiac hypertrophy, the levels of which were dramatically increased in hypertrophic heart tissue and cardiomyocytes. Knockdown of ROR could effectively attenuate the prohypertrophic response via promotion of miR-133 expression (which acted as a ceRNA) and further decreasing the B type natriuretic peptide (BNP) level. LncRNA H19, another negative regulator, was found to protect cardiomyocytes from phenylephrine-stimulated hypertrophy via targeting miR-675. Overexpression of H19 can reduce the increase of cell size and prohypertrophic gene levels, while inhibition of miR-675 abolished the protective effect. In TAC-operated mice model, lncRNA Plscr4 forced expression was indicated to attenuate cardiac hypertrophy by sponging miR-214 and further upregulating mitofusin 2 (Mfn2) expression, a critical modulator of mitochondrial homeostasis. Furthermore, lncRNA MIAT was also verified to participate in the progression of AngII-induced H9C2 cell hypertrophy in vitro by serving as a ceRNA of miR-150.

#### 3.2.3. LncRNAs in Myocardial Electrical Remodeling

Up to date, not many studies have been done on lncRNA-related myocardial electrical remodeling. Li et al. conducted a research by using RNA-seq technique in rabbit models with atrial fibrillation (AF), and the lncRNA expression profiles of right atria were investigated. They identified a total of 99,843 lncRNAs, among which TCONS_00075467 was the selected player. TCONS_00075467 could sponge miRNA-328 both in vitro and in vivo to regulate the gene CACNA1C expression, prolonging the effective refractory period and increasing the action potential duration [70]. MALAT1 was also indicated in regulation of electrical activity by signaling to miR-200c in cardiomyocytes from arrhythmic rats model, and it presented overexpression state. Knockdown of MALAT1 could markedly increase transient outward potassium current and Kv4.2/Kv4.3 channel proteins expression, affecting the outcome of arrhythmias [71].

### 3.3. LncRNAs in Cardiac ECM Remodeling

The cardiac ECM not only provides mechanical support for myocardium, but also transduces essential molecular signals to regulate myocardial function. Dynamic ECM changes happened during myocardial ischemia or chronic volume and pressure overload and ultimately progressed to HF. Cardiac fibroblasts largely drove these pathological processes. Meanwhile, lncRNAs are important regulators of cardiac fibroblasts (CFs) biology. So far, several lncRNAs have been confirmed in cardiac fibrosis and ECM remodeling. By RNA-seq analysis on cardiac samples from ischemic cardiomyopathy patients, Huang et al. found a series of 35 lncRNAs that exhibit strong positive correlation with ECM protein-coding genes. The 5 screened lncRNAs (e.g., n379599, n379519, n380433, n384640, and n410105) by loss- and gain-of-function studies modulated the ECM gene expression, mainly dependent on TGF-*β* pathway [72]. Additionally, in Qu et al.'s study, lncRNAs profile of peri-infarct tissue in mice was checked for bioinformatic analysis, and they found that 263 lncRNAs were significantly upregulated and 282 were downregulated. Among them, NONMMUT022554 was identified as the top-ranked lncRNA positively correlated with genes which were strongly involved in the ECM-receptor interactions [73]. Except for omics study, certain lncRNAs such as MALAT1, wisper, MEG3, H19, and GAS5 were also published to take part in fibrosis and remodeling. In the study performed both in vivo with MI mice model and in vitro with CFs isolated from newborn pups, Huang et al. found that MALAT1 was specifically upregulated in MI heart and in AngII-stimulated CFs; knockdown of MALAT1 could alleviate cardiac fibrosis post-MI and AngII-induced fibroblast proliferation and collagen synthesis by suppressing TGF-*β*1 activity [74]. Wisp2 super-enhancer–associated RNA (Wisper) was another CF-enriched lncRNA that aggravated cardiac fibrosis after injury. Loss-of-function approach in vitro suggested it largely took part in CF proliferation, migration, and survival. Silencing Wisper in vivo in accordance alleviated MI-induced fibrosis and cardiac dysfunction; the regulation of lysyl hydroxylase 2 expression and collagen cross-linking was the possible mechanism [75]. MEG3, mostly expressed by CFs, was found to promote matrix metalloproteinase-2 (MMP-2) production both in vitro and in vivo, thus inducing increased cardiac fibrosis and impaired diastolic performance, silencing of which reversed the effects [76]. The functional validation study discovered that H19 knockdown enhanced the antifibrotic effect of miR-455, decreased the connective tissue growth factor (CTGF) expression, and further inhibited fibrosis-associated protein synthesis, thereby revealing a vital function of the H19/miR-455/CTGF axis in cardiac fibrosis [13]. Furthermore, a suppressive lncRNA in cardiac fibrosis termed GAS5 was also reported. GAS5 was lowly expressed in activated CFs, overexpression of which could inhibit CF proliferation by negatively regulating miR-21 targeting PTEN expression, as it also suggested a potential therapeutic target for fibrosis [77].

## 4. Therapeutic Targeting of LncRNA in Cardiac Remodeling

Cardiac remodeling is a chronic progressive process accompanied by abnormality of cell behaviors, remodeling of matrix components, and impairment of organ function. Certain pharmacological agents or applications of stem cell transplantation and cardiac/remote ischemic conditioning have been found as effective intervention strategies [6, 7], while the exact mechanisms and targets were not so clear. With the advanced knowledge of lncRNAs, the pathogenesis of cardiac remodeling was reinterpreted from the perspective of epigenetics. As mentioned above, numerous studies have confirmed the critical roles of lncRNAs in remodeling by loss- and gain-of-function experiments and have also presumed their potentials as therapeutic targets. According to current data, atorvastatin application has been demonstrated to protect cardiac progenitor cells (CPCs) from hypoxia-induced injury in vitro by inhibiting MEG3 expression, thus providing a clue for lncRNA-related mechanism behind the drug's benefits for MI/HF therapy [78]. Losartan could alleviate Ang II-induced cardiac fibrosis via reversing the downregulation of lncRNA-NR024118 and Cdkn1c in adult rats [79]. Beyond that, manipulating the expression of specific lncRNAs with genetic methods (specifically overexpressed or knockdown) has been largely carried out in animal models, being confirmed to regulate remodeling such as myocardial inflammation, apoptosis, and hypertrophy [55, 57, 67]. Although, theoretically, to modulate lncRNAs' expression specifically—elevating the profiles downregulated or decreasing the ones upregulated in different settings of disease—is an embodiment of precision therapy, a challenge is that the existing research mode can not be completely transformed into clinical trials. On the way to the goal, many questions persist. First, the lncRNAs are poor sequence conservation, and it might be crucial to identify the detailed single-cell or single-molecule profiling at different stages and backgrounds. Second, lncRNAs are usually short-life and damage-prone, and in order to achieve targeted delivery and long-term effectivity, some exogenous gene delivery vectors (e.g., exosomes, nanoparticles, and liposomes) or CRISPR–Cas9 genome editing technology might be required in in vivo experiments, and what followed is how to determine the delivery timing, pattern, concentration, etc.

Until now, there is no clinical trial which has been performed. In the whole noncoding RNA field, anti-miR-122 have finished a phase II clinical trial for treatment of hepatitis C virus infection, which suggested the potential therapeutic role of ncRNAs [80, 81]. Future work would focus on deeper understanding of lncRNA regulatory network, optimization of delivery techniques, and investigation of the interplay of lncRNAs with known beneficial/harmful signaling pathways so as to promote rapid clinical transformation of lncRNAs and their connections with cellular therapy or cardiac regeneration therapy.

## 5. Conclusions

LncRNAs have shown strong regulatory effects in pathological cardiovascular remodeling; therefore, to manipulate lncRNA expression specifically is of promising therapeutic potential. Up to date, most of the available data were in in vitro findings or in animal models, and no clinical trial has been published. In the future, with the development of RNA-seq and gene transfection technology, more lncRNAs linked with cardiac remodeling would be discovered. In addition, targeted delivery of lncRNAs by exosomes or other carriers into the infarcted heart or culprit vessel might promote more meaningful clinical translation.

## Figures and Tables

**Table 1:** LncRNAs involved in vascular remodeling.

Type	Cellular origin	LncRNAs	Regulation	Target genes	Related functions	References
Hypertension	VSMC	TUG1	↑	miR-145-5p	promote migration and proliferation of VSMCs	Shi et al.[28]
	VSMC	AK098656	↑	myosin heavy chain-11/ fibronectin-1	promote VSMC phenotypic switch	Jin et al. [29]
	VSMC	TRPV1	↓	PI3K/Akt	inhibit VSMC phenotypic modulation	Zhang et al. [30]
	VSMC/EC	GAS5	↓	*β*-catenin	affect endothelial activation proliferation, andVSMC phenotypic conversion	Wang et al. [31]
	VSMC	Giver	↑	Nr4a3	promote oxidative stress, VSMC proliferation and vascular inflammation	Das et al. [32]
	VSMC	Lnc-Ang362	↑	miR221/222	promote VSMC proliferation	Leung et al. [33]

Atherosclerosis	VSMC	H19	↑	ACP5 Wnt/*β*-catenin	promote VSMC proliferation and inhibit apoptosis	Huang et al. [9] Zhang et al. [11]
	VSMC/EC/ Macrophage	TUG1	↑	miR-21/PTEN miR-133a miR26a/FGF1	promote VSMC proliferation, facilitate EC/ macrophage apoptosis	Li et al. [37] Chen et al. [38] Zhang et al. [39]
	VSMC	UCA1	↓	miR-26a/PTEN	suppress VSMC proliferation	Tian et al. [40]
	VSMC	MEG3	↓	miR-26a/Smad1	suppress VSMC proliferation	Bai et al. [41]
	VSMC	APPAT	↓	miR-647, miR-135b	affect VSMC phenotype shift	Meng et al. [14]
	VSMC	lincRNA-p21	↓	P53	regulate neointima formation, VSMC proliferation and apoptosis	Wu et al. [42]
	VSMC	Neat1	↑	SM-contractile gene	modulate VSMC phenotype conversion	Ahmed et al. [43]

Arterial aneurysm	VSMC	HIF1A-AS1	↑	caspase-3	regulate VSMC apoptosis	He et al. [47]
	VSMC	lincRNA-p21	↑	TGF-*β*1 signaling	hinder proliferation and promote apoptosis of VSMCs	Hu et al. [48]

VSMC: vascular smooth muscle cell; EC: endothelial cell; TUG1: taurine upregulated 1; TRPV1: transient receptor potential vanilloid type 1; GAS5: growth block specificity 5; Giver: growth factor-and proinflammatory cytokine–induced vascular cell-expressed lncRNA; UCA1: urothelial carcinoma-associated; MEG3: maternally expressed gene 3; APPAT: atherosclerotic plaque pathogenesis associated transcript; lincRNA-p21: long intergenic noncoding RNA-p21; Neat1: nuclear paraspeckle assembly transcript 1; HIF1A-AS1: HIF1 alpha-antisense RNA1; ACP5: acid phosphatase 5; SM: smooth muscle.

**Table 2:** LncRNAs involved in myocardial remodeling.

Type	Cellular behavior	LncRNAs	Regulation	Target genes	Related functions	References
Post-ischemia/hypoxia myocardial remodelling	Autophagy	Neat1	↑	Foxo1	activate autophagy, aggravate CM injury	Ma et al. [49]
		AK139328	↑	miR-204-3p	activate autophagy, aggravate CM injury	Yu et al. [50]
		APF	↑	miR-188-3p	induce autophagic cell death	Wang et al. [51]
		CAIF	↓	p53	inhibit autophagy	Liu et al. [52]
		AK088388	↑	miR-30a	induce autophagic injury	Wang et al. [53]

Post-ischemia/hypoxia myocardial remodelling	Apoptosis	CARL	↓	miR-539	suppress mitochondrial fission and apoptosis	Wang et al. [54]
		MALAT1	↑	miR-203	exacerbate inflammation and apoptosis	Wang et al. [55]
		HOTAIR	↓	miR-125, miR-1	inhibit apoptosis	Li et al. [56] Gao et al. [57]
		UCA1	↑	unspecified	suppress ER-stress and apoptosis	Chen et al. [58]
		XIST	↑	miR-130a-3p	facilitate apoptosis, inhibit proliferation	Zhou et al. [59]

Post-ischemia/hypoxia myocardial remodelling	Necrosis	NRF	↑	miR-873	facilitate necrosis	Wang et al. [60]
		H19	↑	miR-103/107	promote necrosis	Wang et al. [61]

Myocardial hypertrophy	Hypertrophy	Mhrt	↓	Brg1	inhibit cardiac hypertrophy	Hang et al. [62] Han et al. [63]
		Chast	↑	Plekhm1	hinder autophagy and facilitate hypertrophy	Viereck et al. [64]
		CHRF	↓	miR-489	anti-hypertrophic	Wang et al. [65]
		ROR	↑	miR-133	promote hypertrophy	Jiang et al. [66]
		H19	↓	miR-675	protect CMs from hypertrophy	Liu et al. [67]
		Plscr4	↑	miR-214	attenuate cardiac hypertrophy	Lv et al. [68]
		MIAT	↑	miR-150	promote hypertrophy	Zhu et al. [69]

Myocardial hypertrophy	Electrical remodeling	TCONS_00075467	↓	miRNA-328	affect the effective refractory period, increase the action potential duration in AF	Li et al. [70]
		MALAT1	↑	miR-200c	regulate transient outward potassium current	Zhu et al. [71]

Neat1: nuclear-enriched abundant transcript1; APF: autophagy promoting factor; CAIF: cardiac autophagy inhibitory factor; CARL: cardiac apoptosis-related lncRNA; MALAT1:metastasis-associated lung adenocarcinoma transcript 1; HOTAIR:HOX antisense intergenic RNA;UCA1:urothelial carcinoma-associated; XIST:X-inactive specific transcript; Mhrt: myosin heavy chain associated RNA transcripts; Chast: cardiac hypertrophy-associated transcript; Plekhm1:pleckstrin homology domain-containing protein family M member 1; CHRF: cardiac hypertrophy-related factor; MIAT: myocardial infarction–associated transcript; Foxo1:forkhead box protein O1;ER:endoplasmic reticulum; CM: cardiomyocyte; AF: atrial fibrillation.

## Data Availability

The materials in this manuscript are available from the corresponding author on reasonable request.
